# Increasing hepatitis virus screening uptake at worksites in Japan using nudge theory and full subsidies

**DOI:** 10.1186/s12199-021-00940-6

**Published:** 2021-02-01

**Authors:** Jun Fukuyoshi, Masaaki Korenaga, Yui Yoshii, Lek Hong, Soichiro Kashihara, Byron Sigel, Toru Takebayashi

**Affiliations:** 1 Department of Preventive Medicine and Public Health, School of Medicine, Keio University, Shinanomachi, Tokyo, 160-8582 Japan; 2 Hepatitis Information Center, The Research Center for Hepatitis and Immunology, National Center for Global Health and Medicine, Ichikawa, Chiba, 272-8516 Japan; 3 Department of Social and Preventive Epidemiology, School of Public Health, Graduate School of Medicine, Tokyo University, Hongo, Tokyo, 113-0033 Japan; 4 Department of Policy Management, Keio University, Fujisawa, Kanagawa 252-0882 Japan; 5 Cancerscan, Co., Ltd., Shinagawa, Tokyo, 141-0031 Japan; 6 School of International Health, Graduate School of Medicine, Tokyo University, Hongo, Tokyo, 113-0033 Japan

**Keywords:** Nudge, Behavioral science, cancer screening, client reminder, Hepatitis virus screening, Hepatitis at worksites, Message simplification

## Abstract

**Background:**

Despite the importance of hepatitis screening for decreasing liver cancer mortality, screening rates remain low in Japan. Previous studies show that full subsidies increase screening uptake, but full subsidies are costly and difficult to implement in low-resource settings. Alternatively, applying nudge theory to the message design could increase screening at lower costs. This study examined the effects of both methods in increasing hepatitis virus screening rates at worksites.

**Methods:**

1496 employees from a Japanese transportation company received client reminders for an optional hepatitis virus screening before their general health checkups. Groups A and B received a client reminder designed based on the principles of “Easy” and “Attractive,” while the control group received a client reminder not developed using nudge theory. Additionally, hepatitis virus screening was offered to the control group and group A for a co-payment of JPY 612, but was fully subsidized for group B. The hepatitis virus screening rates among the groups were compared using a Chi-square test with Bonferroni correction, and the risk ratios of group A and group B to the control group were also calculated. To adjust for unobservable heterogeneity per cluster, the regression analysis was performed using generalized linear mixed models.

**Results:**

The screening rate was 21.2%, 37.1%, and 86.3% for the control group, group A, and group B, respectively. And the risk ratio for group A was 1.75 (95% confidence interval [CI] 1.45–2.12) and that of group B was 4.08 (95% CI 3.44–4.83). The parameters of group A and group B also were significant when estimated using generalized linear mixed models. However, the cost-effectiveness (incremental cost-effectiveness ratio (ICER)) of the nudge-based reminder with the full subsidies was lower than that of only the nudge-based reminder.

**Conclusions:**

While fully subsidized screening led to the highest hepatitis screening rates, modifying client reminders using nudge theory significantly increased hepatitis screening uptake at lower costs per person.

## Introduction

Liver cancer is one of the leading causes of cancer deaths worldwide [1]. In Japan, an estimated 29,000 people died of liver cancer in 2017 [2]. Furthermore, approximately 70% of liver cancer cases in Japan develop from an infection caused by the hepatitis B virus (HBV) or hepatitis C virus (HCV) [3]. These patients, especially those who are infected with HCV, can be cured with minimal adverse effects using direct-acting antivirals (DAAs) [4]. Thus, the early detection of the hepatitis virus infection by screening is a major public health goal.

To promote hepatitis screening, the Japanese government enacted the Basic Act on Hepatitis Countermeasures in 2009, encouraging municipalities to provide hepatitis virus screening for citizens over 40 years of age [5]. As a result, hepatitis virus screening rates at the municipal level increased. However, hepatitis virus screening rates at worksites remain low in Japan. A previous study surveying 2420 employees showed that only 13.8% had undergone hepatitis screening [6].

Previous studies suggest that providing cancer screenings free of charge could increase screening rates [7]. For example, in Japan, eliminating cost significantly increased screening rates for cervical cancer screening [8]. However, even if free screening is effective, this can add substantial costs to providers as well as employers. Alternatively, applications of nudge theory tend to be low-cost solutions [9], and substantial evidence demonstrates its effectiveness in the realm of cancer screenings [10–12]. Nudge theory, as defined by Thaler and Sunstein, “is any aspect of the choice architecture that alters people’s behavior in a predictable way without forbidding any options or significantly changing their economic incentives” [13]. To illustrate, in Hachioji city, Japan, the city office developed client reminders based on nudge theory and sent to non-takers of colorectal cancer (CRC) screening. These reminders focused on (i) readability and (ii) the low co-payment for screening due to subsidies [14]. This nudge-theory-based reminder led to a CRC uptake of 26.8%, exceeding initial expectations of 19%. Nudge theory could change behavior by modifying the perception of the screening without offering significant financial incentives. Therefore, it is important to compare not only the effectiveness but also the cost-effectiveness of these interventions in promoting hepatitis screening.

Currently, few studies aim to promote the hepatitis virus screening rates at worksites. For many small- to medium-sized companies in Japan, the Japan Health Insurance Association (JHIA) covers the healthcare costs for workers. As of 2015, 17 million workers over the age of 40 were enrolled [15]. This study examines the effects of (i) making screenings free of charge and (ii) applying nudge theory to the design of client reminders on hepatitis virus screening among members of JHIA. Given the large number of premium holders under JHIA, these results could significantly contribute to a reduction in patients with liver cancer in Japan.

## Methods

### Setting

As a member of JHIA, the transportation company provided general health checkups to 12,450 employees as mandated by the Industrial Safety and Health Act [16]. Hepatitis virus screening was offered as an optional test for JPY 612 (US$6) during these general checkups. From 2015 to 2016, a trial program was conducted and designed by the transportation company to increase the uptake of hepatitis virus screening among its employees, who were mostly truck drivers.

### Procedure

Participants were included if they (i) applied and attended the general health checkups conducted in July 2015 and January 2016 and (ii) were 35–74 years old. 1496 employees met the inclusion criteria.

**Fig. 1:**
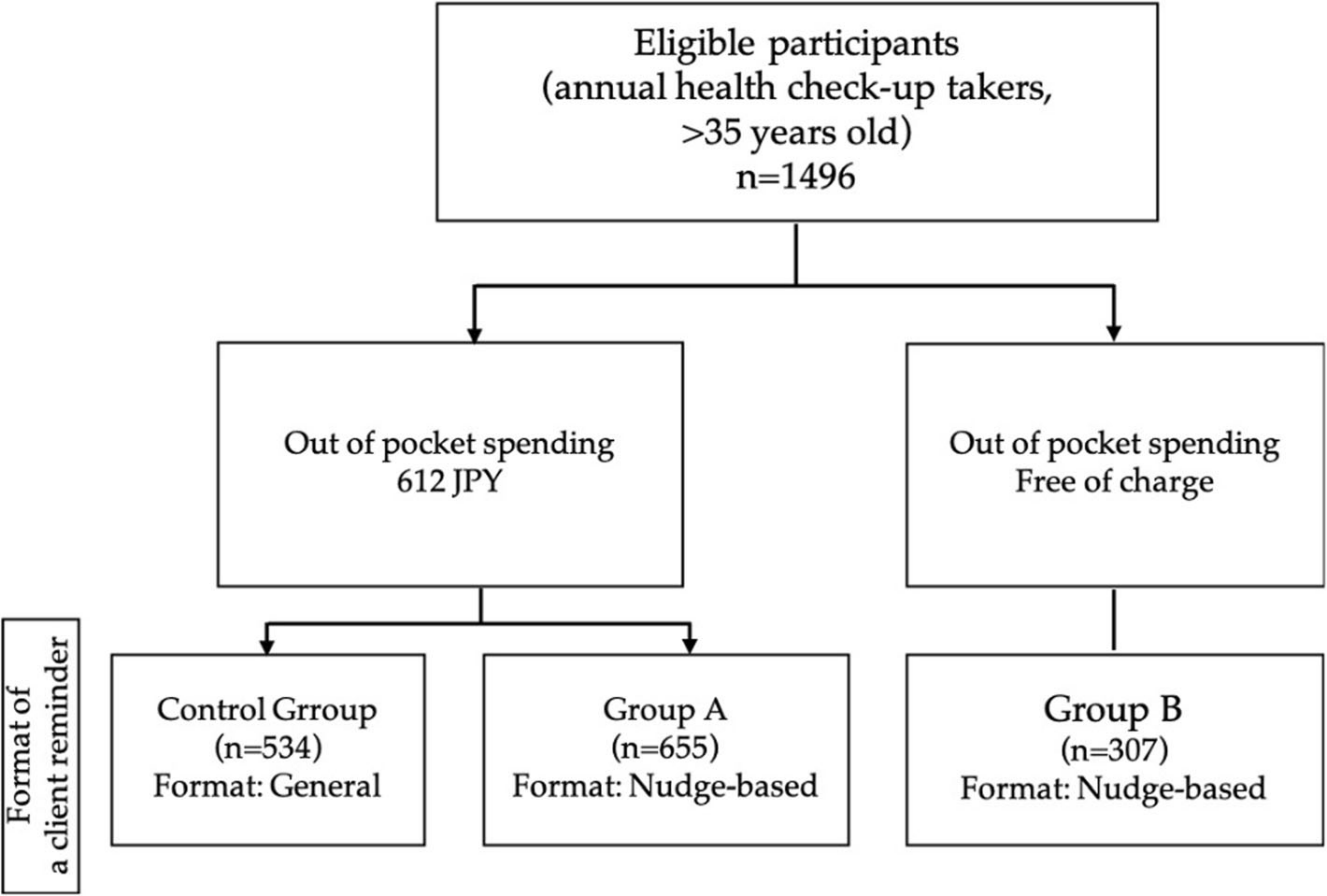
Flow diagram of the trial process

The company designed and executed a cluster-randomized trial, in which the thirteen tracking stations located in urban cities were randomly assigned to control group, group A, or group B (Fig. 1). The employees received different types of client reminders depending on the tracking stations they worked at. Client reminders were sent a few weeks before the general health checkups. Information on the homogeneity among the groups is provided in the results.

### Intervention

**Fig. 2:**
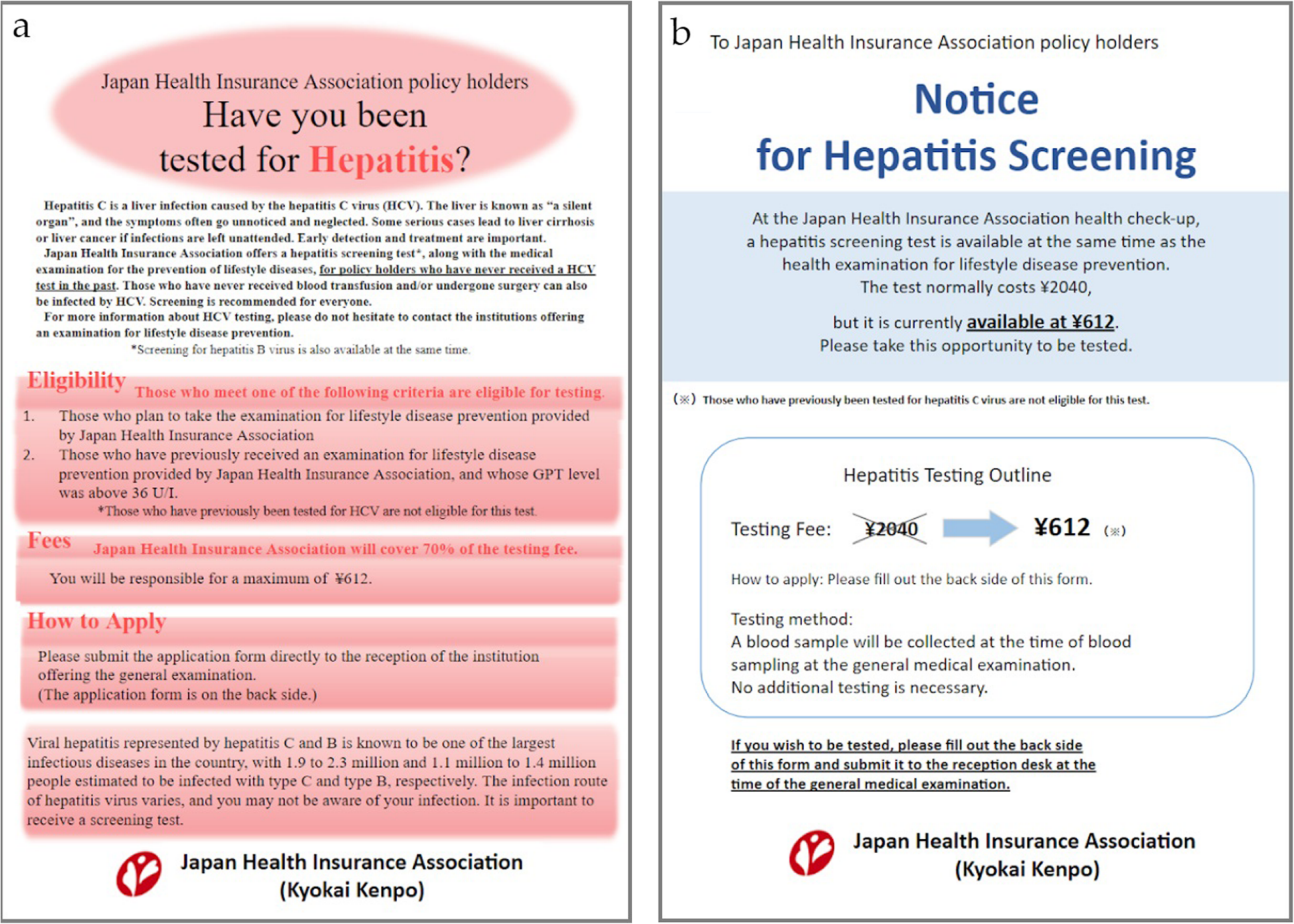
Client reminders (translated). **a** Control group client reminder. **b** Group A client reminder

The control group (*n* = 534) received the client reminder created without applying nudge theory (Fig. 2a). While this reminder included detailed information, including the risks and benefits of undertaking hepatitis virus screening, the reminder was wordy (342 words) and difficult to read.

Group A (*n* = 655) received the client reminder designed using nudge-theory (Fig. 2b), specifically the “EAST” framework [17] created by the UK’s Behavioural Insights Team (BIT). According to BIT, the four principles for effective behavior change are as follows: easy, attractive, social, and timely. Specifically, our study focused on the principles of easy and attractive. To simplify the message, as recommended by the BIT executive report, information on the risks of hepatitis and the benefits of hepatitis virus screening was removed, reducing the word count to 152 words [17]. Also, the discounted cost was emphasized by striking out the original cost of screening (JPY 2040) and using capital letters for the new cost (JPY 612).

### Hepatitis virus screening free of charge

**Fig. 3:**
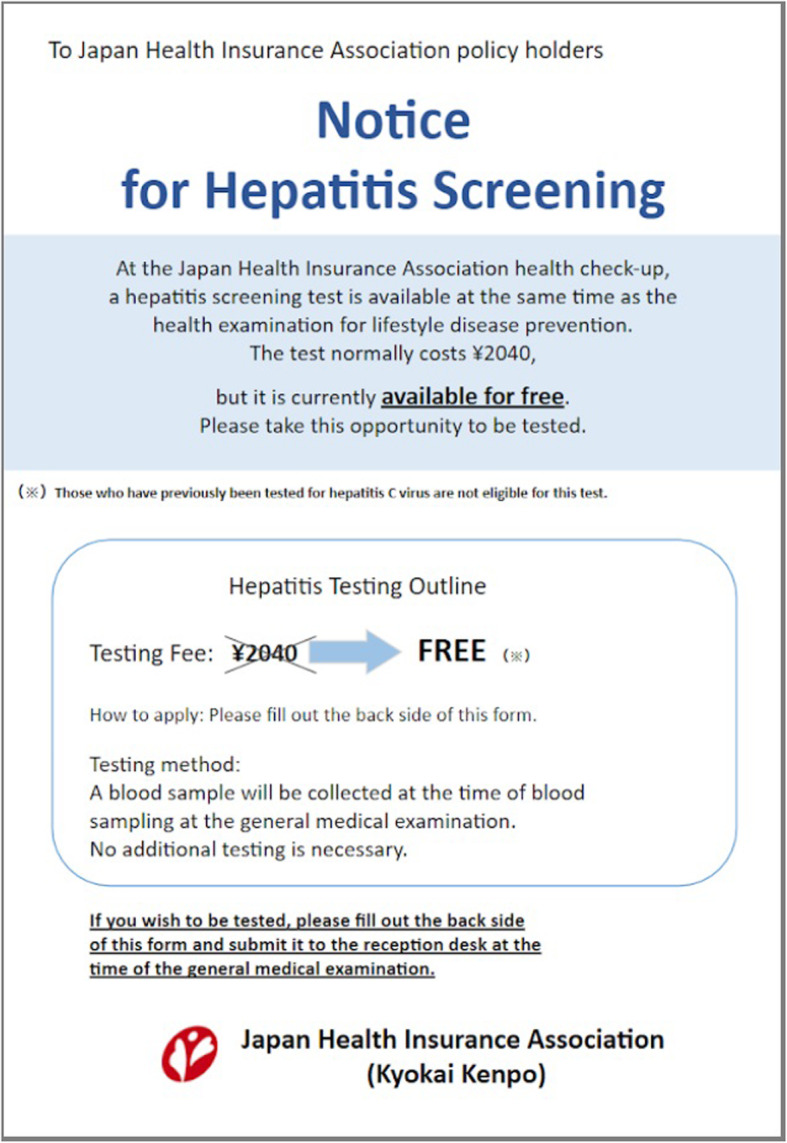
The client reminder for group B

Although the hepatitis virus screening cost of the control group and group A was JPY 612, group B (*n* = 307) was offered the hepatitis virus screening free of charge (shown in Fig. 3).

For group B, the reminder was written in the same format as that of group A, except that the hepatitis virus screening was indicated as free of charge (Fig. 3).

### Data collection and measures

#### Primary outcome

The primary outcome was hepatitis virus screening rate. The healthcare provider checked the number of employees who had undergone the hepatitis virus screening using a standard record-keeping process.

#### Statistical analysis

We described the sex and age distributions of the participants, and we performed a chi-squared test to measure any differences among intervention groups. We quantified the difference in hepatitis virus screening among groups using a chi-squared test with a Bonferroni correction. These analyses were performed using Python 3.6 (The Python Software Foundation, Delaware, USA). Also, we calculated risk ratios. In addition, a regression analysis was performed using generalized linear mixed models to see if significant differences exist even after adjusting for unobservable heterogeneity per cluster. Calculation of risk ratios and the regression analysis were performed using R 4.0.2 (R Foundation for Statistical Computing, Vienna, Austria).

#### Economic analysis

Cost-effectiveness was calculated using the incremental cost-effectiveness ratio (ICER), expressed in terms of JPY per one additional person screened.

$$ \mathrm{ICER}=\frac{\mathrm{Difference}\ \mathrm{in}\ \mathrm{costs}\ \mathrm{between}\ \mathrm{the}\ \mathrm{control}\ \mathrm{group}\ \mathrm{and}\ \mathrm{the}\ \mathrm{in}\mathrm{tervention}\ \mathrm{group}\mathrm{s}\ \left(\mathrm{group}\ \mathrm{A}\ \mathrm{or}\ \mathrm{group}\ \mathrm{B}\right)}{\mathrm{Difference}\ \mathrm{in}\ \mathrm{number}\ \mathrm{of}\ \mathrm{screening}\ \mathrm{takers}\ \mathrm{between}\ \mathrm{the}\ \mathrm{control}\ \mathrm{group}\ \mathrm{and}\ \mathrm{the}\ \mathrm{in}\mathrm{tervention}\ \mathrm{group}\mathrm{s}\ \left(\mathrm{group}\ \mathrm{A}\ \mathrm{or}\ \mathrm{B}\right)} $$

Costs include the design and printing fees for the client reminder, and, in the case of group B, the cost of making screening free of charge.

## Results

### Baseline characteristics of general health checkup takers

Table 1 presents the baseline demographic characteristics of the three study groups. No significant differences among the groups were observed by age and gender.

**Table 1:** The baseline characteristics of the participants in the general health checkup

	Control group		Group A		Group B		***p*** value*
	***n***	(%)	***n***	(%)	***n***	(%)	
**Sex**							
Male	506	94.8%	619	94.5%	291	94.8%	0.97
Female	28	5.2%	36	5.5%	16	5.2%	
**Age**							
30s	73	13.7%	93	14.2%	47	15.3%	0.24
40s	271	50.7%	308	47.0%	130	42.3%	
50s	144	27.0%	189	28.9%	97	31.6%	
60s	40	7.5%	51	7.8%	31	10.1%	
70s	6	1.1%	14	2.1%	2	0.7%	

*There was no significant difference among the three groups in sex and age proportion by chi-squared test

### Effectiveness of interventions on the hepatitis virus screening rates

**Fig. 4:**
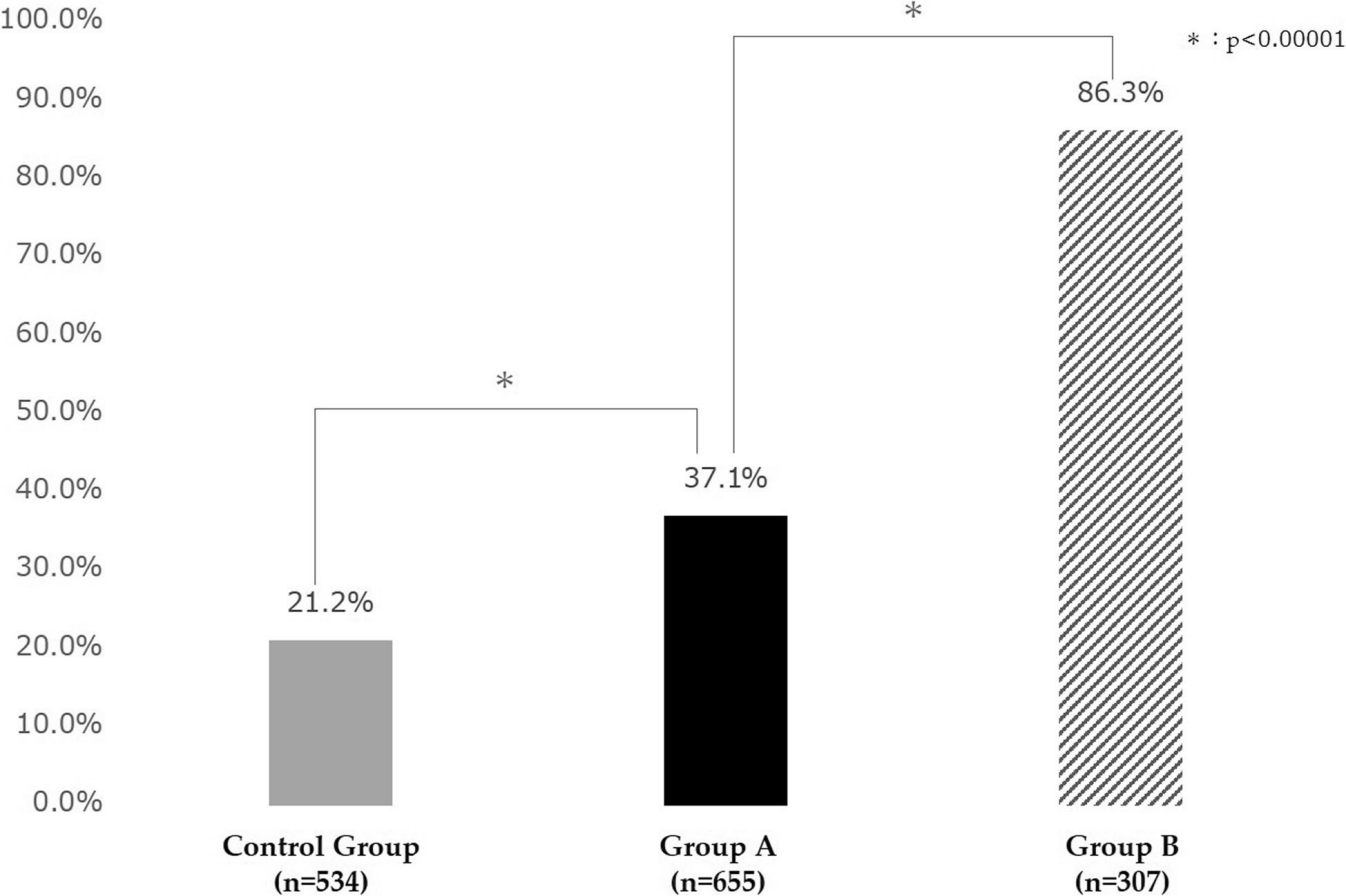
The effectiveness of each client reminder. This figure compares the hepatitis screening rate in the control group in gray (*n* = 534, default design for reminder, screening at usual cost), group A in black (*n* = 655, nudge theory applied to reminder, screening at usual cost), and group B which is shaded (*n* = 307, nudge theory applied to reminder, screening fully subsidized). The screening rate for group A was 15.9% higher than the control group (*p* < 0.00001), and the screening rate for group B was 49.2% higher than group A (*p* < 0.00001). The comparisons were done using a chi-squared test with Bonferroni correction

Figure 4 shows the hepatitis virus screening rates by intervention. While the hepatitis virus screening rate among the control group was 21.2%, group A (who received the nudge-based client reminder) had a 15.9% higher screening rate at 37.1%. The largest difference was observed between group A and group B, where the hepatitis virus screening rate for group B (who were offered the hepatitis virus screening free of charge) was 49.2% higher at 86.3%. The effect of the intervention, expressed as the risk ratio to the control group, was 1.75 (95% CI 1.45–2.12) for group A and 4.08 (95% CI 3.44–4.83) for group B.

Also, to examine whether significant differences exist even after adjusting for unobservable heterogeneity per cluster, a regression analysis was performed using generalized linear mixed models. Table 2 shows the estimation results of the generalized linear mixed models. The variables “Group A” and “Group B” represent the effect of each intervention. The parameters were significantly positive for both group A and group B (both *p* < 0.001), even after adjusting for heterogeneity due to clusters.

**Table 2:** The result of regression using the generalized linear mixed models

	*β*	SE	95% CI	*p* value
Intercept	− 2.241	0.437	(− 3.121, − 1.391)	< 0.00001
Group A	1.109	0.312	(0.480, 1.812)	0.0004
Group B	3.344	0.372	(2.595, 4.170)	< 0.00001
Female	1.211	0.265	(0.696, 1.737)	< 0.00001
Age	0.015	0.008	(0.000, 0.030)	0.0489

*n* = 1496. SE is standard error, and CI is confidence interval

### Cost-effectiveness of each intervention on hepatitis virus screening rates using the ICER calculation

Table 3 shows the cost-effectiveness of each intervention, where a lower ICER value represents greater cost-effectiveness. While the ICER for group B is JPY 1168.7, the ICER of group A is JPY 172.5.

**Table 3:** The cost-effectiveness of the interventions

		Control	A	B
Cost for design of reminder	(1) Design fee (JPY)	0	20,000	20,000
Cost for all employees	(2) Number of employees in each group	534	655	307
	(3) Printing per person (JPY)	20	20	20
	(4) Subtotal cost ((1) + (2) × (3)) (JPY)	10,680	33,100	26,140
Cost for only screening takers	(5) Number of screening takers	113	243	265
	(6) Subsidization cost for making one screening free of charge (applied only to screening takers) (JPY)	0	0	612
	(7) Subtotal cost ((5) × (6)) (JPY)	0	0	162,180
Total cost	(8) Total cost ((4) + (7)) (JPY)	10,680	33,100	188,320
Effectiveness	(9) Additional cost (JPY)	reference	22,420	177,640
	(10) Number of additional screening takers	reference	130	152
	(11) ICER ((9)/10) (JPY/1 additional screening taker)	reference	172.5	1,168.7

### HBV and HCV screening results

No employee from any of the three groups tested positive for the hepatitis B virus. Additionally, the number of employees from the sample test positive for the hepatitis C virus was 1 in the control group (0.2%), 0 in group A (0%), and 4 in group B (1.3%).

## Discussion

To the best of our knowledge, this is the first study to demonstrate the effect of full subsidies and nudge theory on hepatitis screening rates in Japanese worksites. These findings suggest that (i) making screenings free of charge led to the greatest increase in screening uptake, potentially saving many lives and (ii) modifying client reminders using nudge theory could produce a substantial increase in screening uptake at lower costs, making it a viable option in limited-resource settings.

### Findings

Fully subsidized hepatitis virus screening had the highest screening rate of 86.3%, compared to 37.1% for the nudge-based reminder only and 21.2% for the control reminder. The effect of the intervention, expressed as the risk ratio to the control reminder, was 4.08 (95% CI 3.44–4.83) for full subsidy and 1.75 (95% CI 1.45–2.12) for the nudge-based reminder only. In addition, the parameters were significantly positive for both group A and group B, even after adjusting for heterogeneity due to clusters. Previous studies on cancer screening also showed a rise in screening uptake when fully subsidized [18–20]. However, in our study, the cost of screening was only JPY 612 in the nudge-based client reminder only group, yet screening was 49.2% lower. This suggests that requiring a co-payment, albeit small, could discourage many people from undergoing screening. Therefore, if feasible, removing co-payments could promote screening in hard-to-reach populations.

Our study also suggests that applying nudge theory to client reminders increased the hepatitis virus screening rates in these worksites. In particular, our results suggest that providing too much information might reduce the readability of the message, while using nudge-based reminders may increase screening rates in this context. Likewise, a previous study demonstrated that nudge-based client reminders increased colorectal cancer screening rates [14]. To design the nudge-based reminders in this study, we referred to the EAST framework proposed by the UK BIT in their executive report [17], which consists of four principles: (i) “Easy,” (ii) “Attractive,” (iii) “Social,” and (iv) “Timely”. This study followed the principles of “Easy” and “Attractive”. UK BIT recommends, as part of the “Easy” principle, to simplify messages and make them easy to understand. In line with this recommendation, we reduced the number of words on the client reminder. The “Attractive” principle emphasized the importance of drawing attention to important aspects of a message. To this end, the discounted cost of the screening was made salient by striking out the original cost of screening (JPY 2040) and using capital letters for the new cost (JPY 612).

Although making the hepatitis virus screening free produced the highest hepatitis virus screening rates, this led to additional costs. Thus, we performed an incremental cost-effectiveness ratio (ICER) to evaluate the cost-effectiveness of these interventions. The ICER was JPY 1168.7 and JPY 172.5 for the full subsidies and nudge-based reminder only, respectively. Therefore, simply applying nudge theory could significantly increase hepatitis virus screening at lower costs per person, which is critical in low resource settings where offering free screening is not feasible [21, 22]. This finding has implications even at the policy level. In 2011, the Japanese government decided to offer a free coupon for hepatitis virus screening, spending as much as JPY 3.3 billion yen. However, screening increased only by 446,000 people compared to previous year [23, 24]. If the nudge-based reminder were used, it could have cost substantially less and with better cost-effectiveness.

These results also have implications that could affect social implementation. In our study, the ICER of group A is smaller than that of group B. However, it is possible for this relationship to be reversed if the design costs (more generally speaking, fixed costs) are very large. In fact, for the current sample size, number of screening takers, unit cost of full subsidy, and printing costs, that reversal will happen when the design cost exceeds JPY 914,789. On the other hand, a larger design cost means that the absolute value of the ICER will increase in both groups A and B, which means that the financial burden on the insurer will be greater than in the present case. In light of this, the intervention should be implemented with as little design or fixed costs as possible, and, in such cases, the nudge-based reminder only will most likely be more cost-effective.

Even though there were approximately 17 million JHIA subscribers in 2018, only 2 million people received hepatitis testing by the end of 2018 [25]. Therefore, we estimated the impact of scaling up the nudge-based client reminder intervention to all 17 million members of JHIA subscribers if screening rates for both HBV and HCV increased by 16% as shown in our study:

1. The total cost to send nudge-based client reminders will be 341 million yen (20 yen per person for printing and 20,000 yen for designing).
2. Approximately 16,408 HBV carriers and 8204 HCV carriers could be identified (infection rates among the general population for HBV and HCV are estimated at 0.6% and 0.3%, respectively, according to literature) [26, 27].
3. If all the HCV carriers complete their treatment, an estimated 5824 cases of liver cirrhosis could be prevented (approximately 71% of HCV carriers develop liver cirrhosis [28]).
4. About 1514 liver cancer cases can be prevented (26% of HCV liver cirrhosis cases typically develop into liver cancer [29]).

### Limitations

This research has several limitations. First, this study was conducted on employees who applied for and attended general health checkups. Because of the nature of these participants, hepatitis virus screening behavior might differ from that of the general population or from employees who did not sign up for the general health checkups. Second, the positive rates for HCV (0.2%, 0%, and 1.3% for the control group, group A, and group B, respectively) and the HBV (0% for all three groups) were different from our earlier expectations of 0.6% for HBV, and 0.3% for HCV based on previous studies [26]. However, the limited reliability of our measured positive rates due to a small sample size makes it difficult to draw valid comparisons. Third, the demographic data of the sample population is limited to sex and age because the transportation company is not permitted under the Industrial Safety and Health Act to obtain the results of optional tests since companies could identify employees who tested positive and put them at a disadvantage [16]. Hence, further studies are needed to address these limitations. Fourth, the analysis only included 13 clusters. However, after adjusting for heterogeneity of each cluster, our study found significant intervention effects. Nevertheless, increasing the number of clusters might allow us for more precise estimations of effects.

## Conclusions

Full subsidies led to the highest increase in hepatitis virus screening rates and could encourage hard-to-reach populations to undergo screening. However, when full subsidies cannot be afforded by health insurance associations in Japan, most of which are facing severe financial deficits, revising the message of the client reminder using nudge theory could be a more cost-effective means for increasing hepatitis virus screening rates.

## Data Availability

The datasets used and/or analyzed during the current study are available from the corresponding author on reasonable request.

## Abbreviations

BIT: Behavioural Insights Team
CRC: Colorectal cancer
DAA: Direct-acting antivirals
ICER: Incremental cost-effectiveness ratio
HBV: Hepatitis B virus
HCV: Hepatitis C virus
JHIA: Japan Health Insurance Association
JPY: Japanese Yen
USD: US dollar
